# A US‐Based Epidemiological Analysis Reveals Large Discordance Between Exclusive Dietary Recall‐Based Vegetarian Status Assessment and Self‐Identification in a Nationally Representative Population‐Based Survey

**DOI:** 10.1155/sci5/4542935

**Published:** 2026-10-27

**Authors:** Maximilian Andreas Storz, Roman Huber, Christian Koeder

**Affiliations:** ^1^ Department of Internal Medicine II Centre for Complementary Medicine Medical Center-University of Freiburg Faculty of Medicine University of Freiburg Freiburg 79106 Germany uni-freiburg.de; ^2^ Independent Researcher Ellwangen 73479 Germany

**Keywords:** dietary pattern, dietary recall, epidemiology, health surveys, prevalence, vegetarian

## Abstract

**Purpose:**

The greater the flexibility in definitions and concepts, the less likely research findings are to be true. Vegetarian status assessment in epidemiological research is a matter of ongoing controversy, and reported vegetarian prevalence is highly variable depending on the employed assessment methods. A systematic comparison of these potential vegetarian status assessment methods is currently unavailable.

**Methods:**

Using the National Health and Nutrition Examination Surveys (NHANES) data, we examined the vegetarianism prevalence in the United States (2007–2010) using various approaches, including 24‐h dietary recall data only, self‐identification by participants, and various combinations of these approaches.

**Results:**

Large differences and discrepancies were observed: The estimated vegetarian prevalence based on a single 24‐h dietary recall was roughly 10 times higher than the estimated prevalence based on a combination of both self‐identification and 24‐h dietary recall data (14.28% [95%‐CI: 13.37–15.24] *vs.* 1.1% [95%‐CI: 0.77–1.58]). The estimated vegetarian prevalence based on self‐identification alone was twice as high as compared to a combination of both self‐identification and dietary recall data (2.2% [95%‐CI: 1.85–2.60]).

**Conclusions:**

Our findings suggest that epidemiological studies which do not specifically query vegetarian status (and instead rely exclusively on 24‐h‐dietary recalls) may overestimate vegetarian prevalence and are likely subject to misclassification. While self‐identification alone may also result in vegetarian prevalence inflation, our data suggest that the combination of self‐identification and dietary recall data may deliver the most conservative vegetarian prevalence estimate in this scenario.

## 1. Introduction

There has been a recent surge in scientific articles that examine potential associations between health outcomes and vegetarian status [1–3]. Many of these articles rely on large epidemiological databases and studies which were not explicitly designed to examine vegetarian status [1–3]. As such, these studies did not specifically query whether participants considered themselves vegetarian or not. Instead, these studies either relied on 24‐h dietary recall data (based on a single day or multiple days) or determined vegetarian status based on food frequency questionnaires that covered a certain period prior to the participant interview [1–3].

Vegetarians—*by definition*,—do not consume flesh products (meat, fish, seafood, etc.) and, in the case of lacto‐ovo‐vegetarianism, do not consume animal‐sourced foods other than dairy products and eggs [4]. We argue that classifying individuals as vegetarian based exclusively on dietary recall data is an error‐prone approach, and likely results in an overestimation of the vegetarian prevalence in any given population. This may especially be the case in resource‐deprived settings where individuals may not have the financial means to regularly purchase animal‐sourced products (e.g., “vegetarians of necessity” [5]), thus consuming animal products only rarely (but occasionally). Even in high‐income countries, such as the United States, this approach may fail because an individual who does not consume meat products on a daily basis could be easily misclassified as a vegetarian by chance when relying solely on one or two 24‐h‐dietary recalls.

With reference to the United States, we herein provide an example using data from the National Health and Nutrition Examination Surveys (NHANES) [6]. Between 2007 and 2010, the NHANES actively asked participants whether they considered themselves vegetarians [7–9]. We used that information in conjunction with 24‐h dietary recall data from two days to examine the vegetarian prevalence depending on different vegetarian assessment methods. The underlying hypothesis was that an exclusively dietary recall‐based vegetarian assessment results in a much higher vegetarian prevalence than a vegetarian assessment based on self‐identification and dietary intake data combined.

## 2. Methods

Two NHANES cycles (2007/2008 and 2009/2010) were appended and analyzed. The “diet behavior and nutrition module” in both cycles included the following question: “Do you consider yourself to be a vegetarian?” [9]. Potential answer options included “yes,” “no,” and “don’t know.” No additional explanation or definition for the term “vegetarian” was given. Participants who did not know (*n* = 7 unweighted cases) or who refused to answer (*n* = 2 unweighted cases) were not considered for the present analysis. Mobile examination center data were merged with demographic data (including participants’ age, sex, and ethnicity) and food intake data from two 24‐h dietary recalls [9]. NHANES dietary data stem from the dietary interview component, called What We Eat in America (WWEIA), which was conducted as a partnership between the U.S. Department of Agriculture and the U.S. Department of Health and Human Services [10, 11]. All NHANES examinees were eligible for two 24‐h dietary recall interviews conducted by trained interviewers, and only participants with complete data for both recalls were considered here.

Individual food files, including detailed information about the types and amounts of individual foods reported by each participant were combined. Based on food code numbers [10], two distinct groups were created: (i) one group (“omnivores”) who consumed meat and/or poultry and/or fish and/or mixtures (includes meat [first two digit of USDA Food Codes: 20], beef [first two digit of USDA Food Codes: 21], pork [first two digit of USDA Food Codes: 22], lamb, veal and game [first two digit of USDA Food Codes: 23], poultry [first two digit of USDA Food Codes: 24], organ meats, frankfurters, sausages and lunchmeats [first two digit of USDA Food Codes: 25], fish and shellfish [first two digit of USDA Food Codes: 26], meat, poultry and fish mixtures [first two digit of USDA Food Codes: 27], and frozen meals, soups, and gravies with meat [first two digit of USDA Food Codes: 28]) and (ii) one group who did not report any of these foods (“vegetarians”). This was done for each 24‐h dietary recall separately and for both 24‐h recalls combined.

Further to that, we combined 24‐h dietary recalls with self‐reported vegetarian status and investigated how many self‐identified vegetarians did not consume any of these foods. NHANES‐specific survey weights were considered; a 4‐year survey weight was constructed, and weighted proportions were estimated. All NHANES participants between 2007 and 2010 with available dietary recall data (2 days) and no missing data on vegetarian self‐identification were considered regardless of their age.

Comparability of the employed NHANES items across the two cycles was ensured prior to the analysis. The analysis was performed in Stata (StataCorp. 2025. Stata Statistical Software: Release 19. College Station, TX: StataCorp LLC). Data were visualized using pie charts and alluvial plots [12]. The reliability of all estimated proportions was carefully taken into account [13, 14]. More specifically, a low percentage of vegetarians was expected based on the previous NHANES‐related vegetarian literature [7, 8]. As such, we deemed it important to verify whether the estimated vegetarian subpopulation proportions actually met the National Center for Health Statistics’ standards for survey data [13, 14]. These standards require investigators to take the Korn–Graubard confidence interval, along with confidence interval widths, sample size, and degrees of freedom, to assess the reliability of all proportions (especially the vegetarian subpopulations) [13, 14]. For the latter, we used Ward’s user‐written postestimation command kg_nchs [14].

## 3. Results

The final sample included *n* = 15,489 participants with complete data. Table 1 displays participants’ demographic data.

**TABLE 1:** Sociodemographic data of the examined participants in the NHANES (2007–2010) for the whole sample and depending on self‐identification as a vegetarian.

	Whole sample (*n* = 15,489)	Self‐identified vegetarian: No (*n* = 15,162)	Self‐identified vegetarian: Yes (*n* = 327)
*Sex*			
Male	48.14% (47.26–49.03)	48.48% (47.58–49.38)	33.06% (27.47–39.18)
Female	51.86% (50.97–52.74)	51.52% (50.62–52.42)	66.94% (60.82–72.53)

*Age (years)*	37.89 (37.09–38.69)	37.94 (37.14–38.74)	35.70 (32.86–38.53)

*Race/ethnicity*			
Mexican American	9.61% (7.21–12.69)	9.67% (7.24–12.80)	6.87% (4.74–9.86)
Other Hispanic	5.21% (3.65–7.38)	5.19% (3.62–7.39)	6.02% (4.13–8.71)
Non‐Hispanic White	67.45% (62.34–72.18)	67.66% (62.59–72.35)	58.06% (45.06–70.03)
Non‐Hispanic Black	11.67% (9.69–14.00)	11.78% (9.78–14.13)	6.67% (4.18–10.48)
Other race	6.06% (4.95–7.39)	5.69% (4.65–6.95)	22.36% (13.17–35.37)

Figure 1 shows that only a minor proportion of the population considered themselves vegetarian (2.2% [95%‐CI: 1.85–2.60]; Panel a). When looking at food intake data only, however, those who did not report meat or fish products in the first 24‐h dietary recall accounted for 14.28% (95%‐CI: 13.37–15.24) (Figure 1, Panel b). Looking at the second 24‐h dietary recall day only, the prevalence was almost equally high (13.39% [95%‐CI: 12.47–14.37], Figure 1, Panel c). Combining both 24‐h‐dietary recalls resulted in a vegetarian prevalence of 4% (95%‐CI: 3.48–4.58) (Figure 1, Panel d).

**FIGURE 1:**
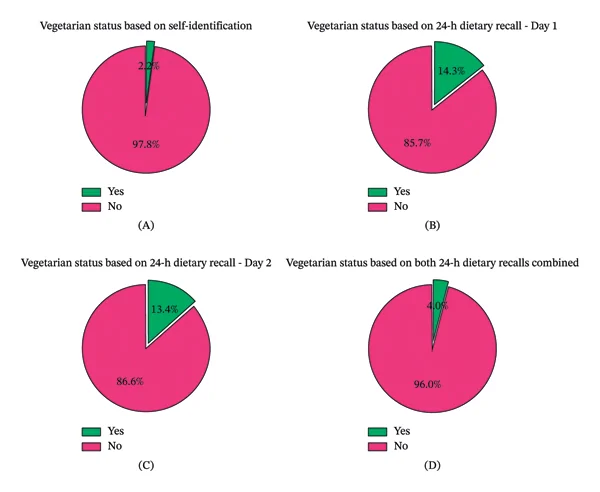
Proportion of vegetarians in the NHANES (2007–2010) depending on self‐identification (Panel A) or single/multiple 24‐h dietary recalls (Panels B–D). This figure is based on *n* = 15,489 NHANES participants with complete dietary recall data for 2 days who either answered “yes” or “no” to the question whether they considered themselves vegetarians or not.

This number was roughly twice as high as the self‐identified vegetarian proportion. When combining self‐identification *and* both dietary recalls (e.g., those who consider themselves vegetarians *and* who did not report meat or fish on both days), the number dropped to 1.1% (95%‐CI: 0.77–1.58). This population accounted for 50% of self‐identified vegetarians (2.2% [95%‐CI: 1.85–2.60]; Panel a). All estimated vegetarian proportions met the National Center for Health Statistics’ standards for survey data [13, 14]. The relevant indicators are presented in Supporting Table 1.

Table 2 shows sociodemographic data of participants who self‐identified as vegetarians but who reported animal‐sourced flesh products in their two dietary recalls. Differences between this subgroup and the self‐identified vegetarian subgroup in general were only observed for race/ethnicity (e.g., a higher share of non‐Hispanic Blacks); however, due to the small sample size and the large confidence intervals, this must be interpreted with great caution.

**TABLE 2:** Sociodemographic data of the examined participants in the NHANES (2007–2010) who self‐identified as vegetarians but who reported animal‐sourced flesh products in their two dietary recalls.

	Self‐identified vegetarian with animal‐sourced foods in the two dietary recalls (*n* = 212)
*Sex*	
Male	32.19% (24.79–40.60)
Female	67.81% (59.40–75.21)

*Age (years)*	38.68 (34.19–43.17)

*Race/ethnicity*	
Mexican American	10.78% (7.07–16.10)
Other Hispanic	9.72% (6.35–14.60)
Non‐Hispanic White	51.56% (38.15–64.92)
Non‐Hispanic Black	10.98% (6.67–17.54)
Other race	16.87% (9.65–27.82)

Figure 2 displays the relationships between self‐identified vegetarian status, dietary recall‐based vegetarian status, and the combination of both in a weighted alluvial plot. This figure illustrates well that some individuals who did not self‐identify as vegetarians would have been accidently (mis)classified as vegetarians if only dietary recalls were considered with reference to the NHANES. Assessment of vegetarian status based on a few dietary recalls thus appears susceptible to misclassification error. This figure also illustrates the relatively high share of individuals who self‐identified as vegetarian but who reported animal‐sourced flesh products in their two dietary recalls (these participants are called semivegetarians in this figure).

**FIGURE 2:**
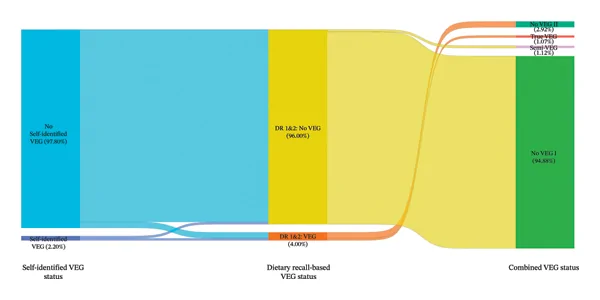
Weighted alluvial plot displaying the relationships between self‐identified vegetarian status, dietary recall‐based vegetarian status, and the combination of both. This figure is based on *n* = 15,489 NHANES participants with complete dietary recall data for 2 days who either answered “yes” or “no” to the question whether they considered themselves vegetarians or not. Created with [12]. Only 1.1% of the sample may be considered “true vegetarians,” as indicated by vegetarian self‐identification and no animal‐sourced flesh food intake in both dietary recalls. Almost 3% of the sample reported no animal‐sourced flesh foods but did not consider themselves vegetarians either. Abbreviations: VEG = vegetarian. SemiVEG = semivegetarian.

## 4. Discussion

This study sought to examine the vegetarian prevalence in the NHANES (2007–2010) depending on various vegetarian status assessment methods. Large differences were observed; the presumed vegetarian prevalence based on a single 24‐h dietary recall was roughly 10 times higher compared to the prevalence based on the combination of both self‐identification and dietary recall data. The discrepancy between a single 24‐h dietary recall and the combination of two days is also notable. The percentage of participants classified as vegetarian based on 24‐h recall data and vegetarian self‐identification (1.1% [95%‐CI: 0.77–1.58]) was much smaller than the self‐identified vegetarian population (2.2% [95%‐CI: 1.85–2.60]; Panel a). Against these findings, it appears noteworthy to cite the seminal work of Vinnari et al. and Gilsing et al., who both suggested that vegetarian self‐identification alone might not be a good approach to estimate vegetarian prevalence [15, 16]. Many individuals who identified themselves as vegetarian did in fact consume animal flesh‐sourced foods. Taking these findings into account, it seems that—in the context of limited dietary recall data—a combination of self‐identification and dietary recall‐based vegetarian assessment is best suited to actually identify “true” vegetarians in the epidemiological analysis.

Given that a representative survey conducted by Harris Interactive suggested a vegetarian prevalence of approximately 3% in the United States in 2009 [17], these findings may be considered benchmarks with important implications for epidemiological research.

In fact, our findings reiterate that caution is warranted when using epidemiological data which do not take self‐identified vegetarian status into account. Not taking self‐identification into account could result in an overestimation of the vegetarian prevalence in any survey with a low number of dietary recall days. For the aforementioned reasons, misclassification risk is nonnegligible and may lead to an inflation of the “vegetarian status” in these surveys when only two recall days are available such as in the NHANES.

Studies which, by definition, include meat or flesh consumption in vegetarianism (e.g., even if reported rarely, as documented earlier [3]) violate the definition of vegetarianism. Moreover, they contravene the fundamental maxim in science that maximum necessary precision, appropriate to the context, must be employed.

Our simple vegetarian prevalence estimate comparison thus has large and important implications:

• Not taking vegetarian self‐identification into account may result in an overestimation of the vegetarian prevalence
• Vegetarian assessment based on self‐identification only may likewise result in an overestimation of vegetarian prevalence, as many self‐identified vegetarians do in fact (occasionally) consume meat
• Vegetarian assessment based on self‐identification *and* dietary recall data may be the most conservative estimate

Against this background, it is prudent to briefly discuss another approach to identify vegetarian status. Vegetarian assessment may also be made based on the usage of detailed food frequency questionnaires that retrospectively cover a longer period (e.g., the last 3 months) [18, 19]. Food frequency questionnaires are easy to administer, cost‐efficient, and frequently used in nutritional epidemiology [20, 21]. Yet controversies persist as to whether this approach alone is sufficient. Very recent evidence based on studies from China has raised concerns that food frequency questionnaires not specifically tailored to vegetarian populations are either too brief (e.g., they do not cover enough vegetarian‐specific food items) or too limited in their scope to capture vegetarian status [18]. Again, the combination of food intake data and self‐assessment appears to be the best approach especially when the aforementioned food frequency questionnaires do not cover all food items necessary to classify an individual as vegetarian (e.g., when intake data on fish and seafood is not specifically queried) [18]. Finally, it is noteworthy that special food frequency questionnaires exist for vegetarians and their usage seems advised in the context of vegetarian and vegan diets [22, 23].

The present contribution has several key limitations worth mentioning. Dietary data are frequently subject to recall‐, social desirability‐, and reporting bias, as well as underreporting [24–26], which could have affected the prevalence estimates presented in this article. The NHANES personal is highly trained and specialized, thus reducing the likelihood of investigator bias. Vegetarianism was not clearly defined in the NHANES, neither in the mobile examination center interviewer procedures manual nor in the dietary manual [27]. This may have introduced ambiguity and skewed the results. The NHANES contains only two recall days and no statements can be made whether the results would have looked different upon including additional recalls. Finally, we acknowledge the lack of multivariate models in the present analysis that adjusted for sociodemographic covariates.

While revising this article, the authors became aware of an earlier, non–peer reviewed Faunalytics analysis containing a comparable analytical approach to ours [28]. Statistical methods and vegetarian categorization, however, were different. Beyond relatively comparable point estimates, this study added interval estimates, carefully reviewed the reliability of the estimated proportions using Korn–Graubard confidence intervals [14], included an alluvial plot to interconnect the different vegetarian categories and a sociodemographic profile of those with divergence between self‐identification and food intake. Despite all discussed limitations, we believe that the herein presented results add to the literature on vegetarian diets and vegetarian status assessment.

## 5. Conclusion

We would like to alert the reader that studies that do not specifically query vegetarian status (and instead rely retrospectively on a very limited number of 24‐h‐dietary recalls) may overestimate the actual vegetarian prevalence in a given study sample and could potentially lead to incorrect diet‐health associations.

## Author Contributions

Conceptualization: Maximilian Andreas Storz and Christian Koeder; data curation: Maximilian Andreas Storz; formal analysis: Maximilian Andreas Storz; investigation: Maximilian Andreas Storz and Christian Koeder; methodology: Maximilian Andreas Storz and Christian Koeder; project administration: Maximilian Andreas Storz, Roman Huber, and Christian Koeder; resources: Maximilian Andreas Storz and Roman Huber; software: Maximilian Andreas Storz; supervision: Roman Huber; validation: Maximilian Andreas Storz and Christian Koeder; visualization: Maximilian Andreas Storz; writing–original draft: Maximilian Andreas Storz; writing–review and editing: Maximilian Andreas Storz, Roman Huber, and Christian Koeder.

## Funding

Open Access funding enabled and organized by Projekt DEAL.

## Ethics Statement

The present study is negligible risk research based on the existing collections of nonidentifiable data about human beings. Research was approved by the NCHS Research Ethics Review Board.

## Consent

Written and informed content was obtained by the NHANES.

## Conflicts of Interest

The authors declare no conflicts of interest.

## Supporting Information

Additional supporting information can be found online in the Supporting Information section.

## Supporting information


**Supporting Information** Supporting Table 1: Korn–Graubard confidence intervals, confidence interval widths, sample sizes, and degrees of freedom used to assess the reliability of all vegetarian‐related proportions in the NHANES (2007–2010) based on Ward’s user‐written postestimation command “kg_nchs” [14]. ^∗^ = The effective sample size adjusted for degrees of freedom. All proportions met all presentation standards and were considered reliable.

## Data Availability

Data are publicly available online (https://wwwn.cdc.gov/nchs/nhanes/Default.aspx). The datasets used and analyzed during the current study are available from the corresponding author.
