# Supplementary material for: A US‐Based Epidemiological Analysis Reveals Large Discordance Between Exclusive Dietary Recall‐Based Vegetarian Status Assessment and Self‐Identification in a Nationally Representative Population‐Based Survey
Source: Scientifica (Cairo). 2026 Oct 27;2026:4542935. doi: 10.1155/sci5/4542935 (PMC13711259; doi:10.1155/sci5/4542935)
Supplement: Supplementary file 1 [file SCI5-2026-4542935-s001.docx]

# SUPPLEMENTRAY TABLE

**Supplementary Table 1**

Supplementary Table 1 title: Korn-Graubard confidence intervals, confidence interval widths, sample sizes, and degrees of freedom used to assess the reliability of all vegetarian-related proportions in the NHANES (2007-2010) based on Ward’s user-written postestimation command “kg_nchs” [14]. * = indicates the effective sample size adjusted for degrees of freedom. All proportions met all presentation standards, and were considered reliable

|  | **Proportion** | **95% CI(p)** | **n *** | **95% CI width (relative width (p))** | **95% CI width abs** |
| --- | --- | --- | --- | --- | --- |
| **Self-identified vegetarian**  Yes  No | 0.0220  0.9780 | 0.0184 – 0.0260 0.9740 – 0.9816 | 5,903.03  5,903.03 | 34.8493  0.7826 | 0.0077  0.0077 |
| **Vegetarian status based on self-identification and dietary recall data**  Self-identification: No & Animal-sourced flesh food intake: Yes  Self-identification: Yes & Animal-sourced flesh food intake: Yes  Self-identification: Yes & Animal-sourced flesh food intake: No  Self-identification: No & Animal-sourced flesh food intake: No | 0.9488  0.0112  0.0107  0.0292 | 0.9433 – 0.9539  0.0091 – 0.0137  0.0072 – 0.0153  0.0261 – 0.0326 | 6,888.54  8,353.80  2,770.58  10,725.25 | 1.1128  41.3333  75.2692  22.1320 | 0.0106  0.0046  0.0081  0.0065 |
